# Obesity-related genomic instability and altered xenobiotic metabolism: possible consequences for cancer risk and chemotherapy

**DOI:** 10.1017/erm.2022.22

**Published:** 2022-07-28

**Authors:** Ezgi Eyluel Bankoglu, Helga Stopper

**Affiliations:** Institute of Pharmacology and Toxicology, University of Wuerzburg, Wuerzburg, Germany

**Keywords:** Cancer, Chemotherapy, DNA damage repair, Drug metabolism, Obesity

## Abstract

The increase in the prevalence of obesity has led to an elevated risk for several associated diseases including cancer. Several studies have investigated the DNA damage in human blood samples and showed a clear trend towards increased DNA damage in obesity. Reduced genomic stability is thus one of the consequences of obesity, which may contribute to the related cancer risk. Whether this is influenced by compromised DNA repair has not been elucidated sufficiently yet. On the other hand, obesity has also been linked to reduced therapy survival and increased adverse effects during chemotherapy, although the available data are controversial. Despite some indications that obesity might alter hepatic metabolism, current literature in humans is insufficient, and results from animal studies are inconclusive. Here we have summarised published data on hepatic drug metabolism to understand the impact of obesity on cancer therapy better. Furthermore, we highlight knowledge gaps in the interrelationship between obesity and drug metabolism from a toxicological perspective.

## Introduction

Obesity is one of the most important health problems of our century (Ref. 1). Today, the number one cause of mortality in the world is noncommunicable diseases and the major risk factors underlying noncommunicable diseases are insufficient physical activity, unhealthy diet and as combination of these two factors obesity (Ref. 2).

Susceptibility for many chronic diseases including cancer is increased by obesity (Refs 3, 4). Almost 20% of all cancers are related to obesity and furthermore, being obese is a factor for poor prognosis in several cancers including breast and colorectal cancers (Ref. 5). It is therefore essential to understand how obesity affects cancer risk and its treatment efficiency for a growing number of patients.

The aim of this narrative review is to summarise the existing knowledge as well as to point out knowledge gaps in the field. For this, we have focused on the association between genomic instability, cancer risk and obesity. Furthermore, we discuss the influence of obesity on the outcome of chemotherapy, for which drug kinetics including metabolism is relevant. There are several other obesity-associated factors such as alterations in gut microbiome, hormonal and immune system status, which might be linked to cancer risk as well as influence cancer therapy. However, they are not within the scope of this review.

## Search strategy and selection criteria

We intended to emphasise the relationship between obesity, genomic instability and cancer risk. In addition, we wanted to discuss the effect of being obese as cancer patient and the impact of obesity on cancer chemotherapeutic treatment and cytostatic drug metabolism. Finally, we planned to define knowledge gaps and indicate possible future research directions. The included articles were published from 2000 onwards. In addition, we conducted a manual search on reference lists of relevant articles in order to find additional references.

The following key words were included in our PubMed search: ‘DNA damage, genomic instability, DNA repair, cancer risk, obesity’, ‘drug metabolism, cytochrome P450, pharmacokinetic, cancer therapy, chemotherapy, obesity’. Based on the paper abstracts and availability, suitable publications for this narrative review were selected.

## Influence of obesity on genomic stability

There are several studies which showed elevated DNA damage in obese subjects (Table 1). Most of these studies were conducted with blood samples. Comet assay, micronucleus test and fluorescence staining to determine phosphorylated H2Ax were the most commonly performed endpoints to measure DNA damage in humans.

**Table 1.** Influence of obesity on genomic stability

Study population	Endpoints	Key findings	Reference
Normal-weight controls (*n* = 21), over-weight (*n* = 21) and obese (*n* = 83)	Micronucleus test in lymphocytes and 8-OHdG in plasma	↑ MN in obese versus normal weight and over-weight ↓ 8-OHdG in obese versus normal weight and over-weight	7
Obese (*n* = 45) before and after bariatric surgery at 6 and 12 months	Micronucleus test in lymphocytes	↑MN in obese before bariatric surgery versus after bariatric surgery at 12 months	8
Italian children, normal weight (*n* = 38), over-weight (*n* = 20) and obese (*n* = 61)	Micronucleus test and *γ*-H2Ax foci in lymphocytes	↑ MN and *γ*-H2Ax foci in over-weight and obese children versus normal weight children	86
Normal weight (*n* = 15) and obese (*n* = 23) adolescents	*γ*-H2Ax foci in lymphocytes without or with 0.3 μg/ml mitomycin treatment at 2 and 4 h	↑ Basal *γ*-H2Ax foci in obese versus normal weight ↑ *γ*-H2Ax foci induction following to mitomycin c treatment in obese versus normal weight ↑ Repair of mitomycin c induced *γ*-H2Ax foci in obese	12
Normal weight (*n* = 26) and obese (*n* = 88) women	Alkaline comet assay in lymphocytes	↑ DNA strand breaks in obese versus normal weight women	14
Normal weight control (*n* = 160) and obese (*N* = 160)	Alkaline comet assay in leucocytes	↑ DNA strand breaks in obese versus normal weight	15
Normal weight (*n* = 300) and obese (*n* = 300) women	Alkaline comet assay, FPG comet assay and Endo III comet assay	↑ DNA strand breaks, alkali-labile sites and oxidised bases in obese versus normal weight women	16
Obese (*n* = 41) before and after bariatric surgery at 12 months	Alkaline comet assay and FPG comet assay	↓ DNA strand breaks in obese before versus 12 months after bariatric surgery ↔Net FPG sensitive sites in obese before versus 12 months after bariatric surgery	17
Young healthy individuals (*n* = 99)	Alkaline comet assay, FPG comet assay and 8-oxodG by HPLC-ECD in lymphocytes	Positive correlation between increasing BMI (<24) and oxidised DNA damage by FPG comet assay in females	18
Breast cancer patients (*n* = 70) and control (*n* = 70)	Alkaline comet assay in PBMCs	↑ DNA strand breaks in breast cancer patients versus controls Positive correlation between DNA strand breaks and increasing BMI (<25) in breast cancer patients	19
Healthy volunteers supplemented with micronutrients daily for 6 weeks, healthy volunteers (*n* = 9) unsupplemented	The host cell reactivation assay to determine NER capacity in stimulated PBMCs (400 J/m^2^ UV treatment)	No significant effect of supplementation ↓ NER capacity with increasing age and BMI (<35)	23

Increase (↑), decrease (↓) and no change (↔).

Micronucleus formation is a well-known endpoint for assessment of chromosomal damage. A micronucleus represents a chromosome or chromosomal fragment, which could not integrate into one of the daughter nuclei during mitosis (Ref. 6). An elevated micronucleus frequency in the peripheral blood mononuclear cells of obese subjects has been reported previously (Refs 7, 8, 9). Furthermore, Bankoglu and colleagues have shown a significant reduction in micronucleus frequency of obese subjects following weight loss which was induced by bariatric surgery (Ref. 8). Micronuclei can arise from an aneugenic or a clastogenic effects (Ref. 10). Clastogenic events are associated with DNA double-strand breaks, which in turn lead to the phosphorylation of histone 2Ax (termed *γ*-H2Ax), yielding a sensitive and reliable marker for DNA double-strand break detection (Ref. 11). Scarpato *et al*. (Ref. 9) investigated the DNA damage in peripheral blood mononuclear cells from overweight and obese children compared with normal weight children. Their findings revealed an increase in micronucleated and *γ*-H2Ax positive cells in overweight and obese children compared with normal weight children. In another study, *γ*-H2Ax analysis was performed in peripheral blood mononuclear cells (PBMCs) of obese and normal weight adolescents. The obese adolescents showed a higher frequency of *γ*-H2Ax positive cells compared with the normal weight adolescents (Ref. 12).

The alkaline comet assay can detect single- and double-strand breaks. Modified comet assay versions can be used to measure specific lesions. For example, the detection of oxidised bases is enabled by the addition of formamidopyrimidine DNA glycosylase enzyme (Ref. 13). Most of the studies that investigated the influence of obesity on DNA damage applied the alkaline and FPG comet assay. Using the alkaline comet assay in PBMCs from obese subjects, Włodarczyk *et al*. (Ref. 14) showed an increase in DNA strand breaks compared with cells from normal weight individuals. Bukhari *et al*. (Ref. 15) performed the alkaline comet assay with leucocytes from normal weight and obese subjects and their results indicated a higher DNA damage in leucocytes from obese subjects. Luperini *et al*. (Ref. 16) investigated the DNA damage in morbidly obese women compared with normal weight women by applying the FPG comet assay. Their findings showed a significant increase in DNA strand breaks and oxidised DNA bases in PBMCs of obese women. Also using the FPG comet assay, Bankoglu *et al*. (Refs 8, 17) found a significant reduction in DNA strand breaks but not in oxidative DNA lesions in PBMCs from obese subjects after a significant weight reduction induced by bariatric surgery.

Hofer *et al*. (Ref. 18) analysed the DNA damage in young healthy individuals and their findings showed a positive correlation between oxidative DNA damage measured by formamidopyrimidine DNA glycosylase (FPG) comet assay and body mass index (BMI) values (<24) in females. In another study, the alkaline comet assay was used to measure DNA strand breaks in breast cancer patients and healthy controls and BMI was a confounding factor influencing DNA damage. Interestingly, the DNA damage showed a positive correlation with BMI in breast cancer patients, but a negative correlation in healthy controls. In this study, the BMI was not different between the breast cancer patients and healthy controls and it was within the normal range (Ref. 19).

Obesity has been linked to several factors that can contribute to increased genomic instability. Some of these factors can be listed as chronic inflammation, oxidative stress, altered levels of hormones such as insulin, formation of lipid peroxidation products and advanced glycation products. Setayesh and colleagues (Ref. 20) published a detailed review on the impact of obesity on DNA stability, which provides an excellent overview of the contributing mechanistic factors.

Clearly, obesity is associated with increased DNA damage. However, under normal conditions DNA damage can be repaired efficiently by various DNA repair proteins and pathways. In order to understand the influence of obesity on genomic stability and cancer risk, the elucidation of the influence of obesity on DNA repair is essential. However, few studies regarding DNA repair in obesity in human are available. Wang *et al*. (Ref. 21) identified a set of key drivers and genes which might be related to obesity (defined with several measures such as BMI, waist–hip ratio and computed tomography analysis) and most of these identified genes were important for cell cycle and DNA repair. In another study, Azzarà and colleagues analysed the DNA repair kinetics of mitomycin C-induced DNA damage in phytohaemagglutinin-stimulated peripheral blood lymphocytes by using *γ*-H2Ax staining. The same amount of in vitro mitomycin C treatment yielded a higher *γ*-H2Ax signal for PBMCs from obese adolescents compared with those from normal weight individuals after 2 h of treatment. However, after 4 h, the induced DNA damage was repaired more efficiently in the PBMCs from the obese individuals than in those from the normal weight individuals (Ref. 12).

Nikodemova and colleagues investigated the effect of smoking on transcriptional differences (Ref. 22). Their findings showed gene expression differences between obese and non-obese smokers. In this study, obesity was linked to an altered expression of 29 genes. Among these genes, some were related to DNA damage response and repair, such as p53, RAD51 and GADD45A. This study indicated a possibility for increased susceptibility of obese subjects against environmental exposure. Tyson and colleagues studied the effect of micronutrient supplementation on nucleotide excision repair in PBMCs before and after 6 weeks of supplementation (Ref. 23). The study participants were genotyped for four polymorphisms in NER genes. There was no significant effect of individual polymorphisms or of micronutrient supplementation on NER capacity. However, the authors found a negative correlation between NER capacity and increasing BMI values.

Overall, the published studies indicate a possible modulatory role of obesity on DNA repair activity; however, further research is needed in this area.

## Obesity and cancer risk

Accumulation of DNA damage because of missing or erroneous repair and increased genomic instability are key contributors to cancer development. There are several cohort studies that reported their observations regarding obesity and cancer risk (Table 2). The International Agency for Research on Cancer (IARC) reviewed these epidemiological studies together with studies in experimental animals and published an opinion paper on obesity and cancer in 2016 (Ref. 24). In this, IARC deduced that obesity is a risk factor for several types of cancers (Ref. 24). In the Norwegian Women and Cancer (NOWAC) study, the authors investigated the effect of body weight change and obesity-related cancer risk. They observed an overall increase in obesity-related cancer risk among overweight and obese women. In addition to this, a weight gain >10 kg over 6 years led to an increase in obesity-related cancer risk independent of BMI status at baseline (Ref. 25). Obesity is now widely accepted as one of the preventable cancer risk factors in addition to tobacco smoking (Refs 26, 27, 28). Birks and colleagues (Ref. 29) published a systematic review on the impact of weight loss on cancer incidence and mortality. Their paper showed the beneficial effect of intentional weight loss (including bariatric surgery) on overall cancer risk. In this context, it is important to emphasise the relevance of sustaining an achieved weight loss. Teras *et al*. (Ref. 30) investigated the effect of sustained weight loss on breast cancer in women. Sustained weight loss was defined as 2 or more kg lost in a first interval (5.2 years), which was not regained in the second interval (4 years). Their study showed a reduced breast cancer risk for women who managed to sustain weight loss, whereas women, who lost less than 9 kg and regained most of it had a similar breast cancer risk compared with the group without weight loss (Ref. 30).

**Table 2.** Association between obesity, weight loss and cancer risk

Study population	Cancer type	Key findings	Reference
The Norwegian Women and Cancer population-based cohort study (enrolment from 1991 to 2005, *n* = 138 746; followed up until cancer diagnosis, death or December 2014)	Colon, colorectal, kidney, endometrial, breast, pancreatic cancers	9328 women were diagnosed with obesity-related cancers at average age of 62 ↑ Cancer risk because of obesity for colon, colorectal, kidney, endometrial and postmenopausal breast Strong association with pancreatic cancer and high weight gain (⩾10 kg)	25
A systematic review on the association between the weight loss and cancer incidence or mortality (from publications between 1978 and 2011)	Obesity-related cancers: postmenopausal breast, colon, colorectal, endometrium, kidney, pancreas and oesophagus	↓ Cancer risk in bariatric surgery population ↓ Mortality from all cancers in weight loss categories	29
The Pooling Project of Prospective Studies of Diet and Cancer: More than 180 000 women aged 50 or more years old, who were cancer free at the beginning of follow-up, followed for 8 years for breast cancer	Breast cancer	↓ Breast cancer risk for women with sustained weight loss versus women with stable weight ↓Breast cancer risk for women who lost weight (⩾9 kg)	30

Increase (↑), decrease (↓) and no change (↔).

Bankoglu *et al*. (Ref. 31) investigated the effect of a broad range of BMI on oxidative DNA damage. Their findings supported the possibility of a linear relationship between BMI and oxidative DNA lesions, although the number of subjects between BMI values 30 and 40 was too limited for a final interpretation. If linearity holds true, any amount of weight loss might be beneficial for reduction of the oxidative DNA damage in obese subjects.

In conclusion, weight loss can be beneficial for preventing obesity-related cancer, but the amount of required weight loss, the effect of weight loss methods and the consequences of a partial weight regain have not been fully elucidated.

In addition to endogenous factors, the link between obesity and cancer can also be influenced by exogenous risk factors. Since dietary exposure is one of the most common routes for chemical carcinogens, dietary preferences of individuals can shape the exposure pattern and their individual cancer risk. Metabolic enzymes can either convert a substance to an inactive metabolite and thus help in its elimination or can create reactive intermediates which can exert a toxic or mutagenic effect (Refs 32, 33). Numerous carcinogens are metabolically activated or inactivated. Examples are dietary contaminants such as polycyclic aromatic hydrocarbons, the mycotoxin aflatoxin B1 or nitrosamines (Ref. 34). The possible influence of external exposure and altered xenobiotic metabolism is discussed in the review by Setayesh and colleagues (Ref. 20). From a toxicological perspective, obesity may affect the susceptibility to external exposures via alterations in xenobiotic metabolism. There are numerous publications which found differences in expression and/or activity of metabolising enzymes linked to obesity. We discuss these studies in the section ‘Influence of obesity on pharmacokinetic and dynamic processes’ in the context of the influence of obesity on pharmacokinetic and dynamic processes. However, to understand the possible effect of obesity on susceptibility of individuals to external exposure and the contribution of alterations in xenobiotic metabolism, human biomonitoring studies linking exposure data to cancer incidences and to metabolomics and transcriptomics data might be helpful.

## Possible impact of obesity on cancer therapy

Since obesity is defined as a cancer risk factor, its increase in prevalence will contribute to new cancer cases as well as enhance the number of obese cancer patients (Ref. 35). However, available information regarding the influence of obesity on prognosis and therapy outcome is limited (Table 3). Obesity has a strong association with several pathophysiological conditions, such as increased blood volume, low-grade inflammation, fatty liver, increased cardiac output and increased hepatic blood flow. The influence of these conditions on specific xenobiotic metabolism is not yet clear (Ref. 36). Calle *et al*. (Ref. 37) showed that BMI values equal to or greater than 40 lead to increased cancer mortality after 16 years of follow-up. They estimated that overweight and obesity might contribute with 14% to all cancer deaths in men and with 20% to all cancer deaths in women. For example, obesity is associated with reduced survival in breast cancer. Besides, obesity can lead to a poor prognosis for breast cancer (Refs 38, 39, 40). Among colon cancer patients who received 5-fluorouracil-based adjuvant chemotherapy, obese and underweight but not overweight patients were affected by reduced survival outcome compared with normal weight patients (Ref. 41). In line with this study, the article from Renfro and colleagues revealed the relationship between BMI and colon cancer therapy outcome as J-shaped, with an overall better survival for overweight (non-obese) patients compared with underweight and obese patients with a BMI of 35 or more (Ref. 42). In contrast with these two studies, a better overall survival with higher BMI values was demonstrated for patients with metastatic colorectal cancer. Regarding side effects, higher BMI values were associated with higher risk for nausea, vomiting and peripheral neuropathy, whereas patients with lower BMI values had a higher risk for haematological toxicities (Ref. 43). This different influence of obesity on cancer treatment side effects and treatment outcome is discussed in a recent review by Gallo and colleagues (Ref. 44).

**Table 3.** Impact of obesity on cancer therapy

Study population	Cancer type	Key findings	Reference
Cancer Prevention Study II by American Cancer Society, with a final population 900 053 participants with a follow-up between 1982 and 1998	Oesophageal, stomach, colorectal, liver, gallbladder, pancreatic, lung, melanoma, prostate, bladder, kidney, brain cancers, non-Hodgkin's lymphoma, multiple myeloma, leukaemia, etc.	↑ Mortality because of cancer in both men and women with higher BMI Increased mortality from cancer with higher BMI in men with prostate and stomach cancers and in women with uterus, cervix, ovary and breast cancers	37
Meta analyses on obesity and survival in women with breast cancer	Breast cancer	↓ Survival in obese women with pre- or postmenopausal breast cancer versus normal weight women	40
Meta analyses on obesity and breast cancer survival	Breast cancer	↑ Risk for breast cancer recurrence and related mortality in obese patients	38
Meta analyses on obesity and survival in women with breast cancer	Breast cancer	↓ Survival in obese women with breast cancer versus not obese women with breast cancer	39
Data from 25 291 stage II and III colon cancer patients	Colon cancer	↓ Survival outcome among obese (especially men) and underweight colon cancer patients treated in adjuvant chemotherapy trials	41
Data from 15 936 stage III colon cancer patients in phase III clinical trials	Colon cancer	↓ Survival for the colon cancer patients with underweight and obese (BMI ⩾ 35)	42
Pooled data from 5 clinical trials	Metastatic colorectal cancer	↑ Survival with higher BMI among patients with metastatic colorectal cancer Higher BMI associated with higher risk for nausea, vomiting and peripheral neuropathy	43
Meta analyses on effect of obesity on toxic chemotherapy outcome	Lung cancer, breast cancer, leukaemia, gastrointestinal carcinoma, Hodgkin lymphoma and colorectal cancer	Majority of reported studies did not find a significant difference in therapy side effects between obese and normal weight patients	45
The early breast cancer patients (*n* = 325, 24.5% obese and 33.5% overweight, 42% normal weight)	Breast cancer	No difference in febrile neutropenia rate in obese and overweight versus normal weight	46
The GAIN Study: 3023 patients between August 2004 and July 2008 for comparing dose-dense regimen between obese and normal weight patients	Breast cancer	↑ Risk in obese patients who received chemotherapy according to their actual body surface area for severe side effects	47
Pharmacokinetic of 8 chemotherapeutics were studied in 1206 cancer patients (of 13.4% obese)	Not specified	For cisplatin, paclitaxel and troxacitabine in obese patients: ↑ absolute clearance For doxorubicin in obese patients: ↓ systemic clearance	48
Meta analyses on obesity and risk for cardiotoxicity in breast cancer patients	Breast cancer	↑ Risk for developing cardiotoxicity after anthracyclines and trastuzumab in breast cancer patients with over-weight or obesity	49

Increase (↑) and decrease (↓).

Hourdequin and colleagues performed a meta-analysis comparing obese patients with normal weight patients after a chemotherapeutic drug dosing calculated with actual body weight. Their findings did neither indicate an increased chemotherapy toxicity nor reduced survival outcome in obese patients (Ref. 45). Lote *et al*. (Ref. 46) investigated the side effect febrile neutropaenia in obese women with breast cancer and their findings did not reveal an increased toxicity for obese women. They recommend not to use a reduced chemotherapy dose for obese patients. Opposite to this, the GAIN study showed a higher toxicity of chemotherapy in those obese breast cancer patients, who received a dose which was adjusted to body surface area compared with unadjusted doses and to normal weight patients (Ref. 47). Sparreboom *et al*. (Ref. 48) studied the pharmacokinetics of eight anticancer drugs in lean and obese cancer patients. The drugs were administered intravenously and the dose for each drug was normalised according to body surface area. They found a significant increase in the clearance of cisplatin, paclitaxel and troxacitabine in obese compared with lean patients. However, the clearance of doxorubicin was significantly reduced in obese women, but not in men. This study showed that obesity may alter the pharmacokinetics of chemotherapeutic drugs in a sex-dependent and drug-specific manner. Guenancia and colleagues performed a meta-analysis to investigate the association between obesity/overweight and cardiotoxicity from anthracyclines in breast cancer therapy. Their study showed that being obese/overweight increased the risk of cardiotoxicity after anthracycline treatment (Ref. 49). In another study, the effect of body composition on pharmacokinetics and toxicity of doxorubicin was studied in breast cancer patients. The study results revealed an increased doxorubicin exposure area under the curve (AUC) related to higher intra-abdominal fat content and indicated a higher risk for doxorubicin toxicity for patients with excess body fat. The authors suggested to use the body composition as a better marker for determining doses in chemotherapy than the body surface area (Ref. 50). The dose of chemotherapeutics in cancer therapy is based on existing clinical trials and the calculated body surface area of a patient. However, many oncologists use an ideal body weight to calculate the drug doses in order to avoid possible overdosing and toxicity in obese patients (Refs 51, 52). This might contribute to a reduced survival rate among obese patients. It is increasingly recognised that body surface area or BMI is not an accurate basis for the choice of the treatment doses.

In addition to altered kinetics, the state of obesity is likely to lead to modifications in protein expression and enzyme activity (Refs 53, 54) which might be important for the uptake and transport or for the metabolism and excretion of chemotherapeutic drugs and other xenobiotics.

Understanding the interrelationship between obesity and drug efficiency better is crucial for reducing treatment side effects and mortality in cancer patients as well as for the adaptation of drug treatments to the rising incidence of obesity in general.

## Influence of obesity on pharmacokinetic and dynamic processes

Variabilities in hepatic and extra-hepatic metabolic enzymes can lead to local and systemic toxicity in obese patients after exposure to both chemotherapeutic agents and environmental carcinogens. An altered activity of some drug metabolising enzymes in the obese state has been shown (Refs 53, 54, 55, 56). However, the available information in humans is limited to a few cytochrome P450 enzymes. Because of the limited number of studies in humans, we decided to include the existing rodent studies in this paper (Table 4).

**Table 4.** Influence of obesity on pharmacokinetic and pharmacodynamic processes in rodent obesity models and in humans

Species	Sex	Age	Obesity model	Findings	Reference
Zucker (+/+) rat and obese Zucker (*fa*/*fa*) rat	m	20-week-old	Genetic and HF diet Zucker (+/+) rats were either fed with ND (4.1% fat, ND rats) or with HF diet (8.6% fat, HF rats), obese Zucker (*fa*/*fa*) rats were fed with ND (obese rats) Single-dose chlorzoxazone (CZX, 20 mg/kg intravenous) administered	mRNA expression: *Cyp2e1* ↑ in liver and fat tissue of HF and obese rats and in kidney ↔ Protein expression: CYP2E1 ↑ in liver and fat tissue of HF and obese rats and in kidney ↔ The area under the plasma concentration time curve of CZX: ND > HF > obese microsomal activity of Cyp2e1: obese > HF > ND	57
C57BL/6J mouse (wild type and ob/ob)	m/f	12-week-old	Genetic	mRNA expression: *Ho-1*, *Nqo1*, *Sult2a1/2*, *Cyp4a14* and Cyp2b10 ↑, Cyp2e1 ↓ and *Cyp3a11* ↔ (m); *Cyp2b10*, *Cyp3a11* and *Cyp2e1* ↓, *Nqo1* ↑, *Ho-1*, *Cyp4a14* and *Sult2a1/2* ↔ (f) Protein expression: analysed only for HO-1 (m: ↑ and f: ↓), NQO1 ↑, CYP2E1↓ and CYP3A11↓ (m and f)	58
C57BL/6 mouse	m	25-week-old	HF diet (40% beef tallow), metformin application either intraperitoneal or intravenous	mRNA expression: OCT ↑ protein expression: OCT ↑ The plasma and hepatic concentration of metformin ↑ in obese animals	59
C57BL/6J mouse	m	28-week-old	HF diet (45% fat)	Protein expression: CYP1A2, CYP3A11, UGT1A, UGT2B, SULT1A1 ↔ and SULT2A1 ↓ Enzyme activity: CYP1A2, CYP3A11, total UGT ↑ and total SULT ↓	60
Zucker (+/+) rat and obese Zucker (*fa*/*fa*) rat	m	27-week-old	Genetic Single tacrolimus administration either by intravenous injection or by orally	Protein expression: CYP3A2 ↓ in liver and ↔ in intestine, P-glycoprotein ↔ in liver and ↓ in intestine in obese Zucker rats Blood concentration of tacrolimus and its bioavailability ↑ in obese Zucker rats via both application routes	61
NMRI mouse	m	32-week-old	Monosodium glutamate administration	mRNA expression: *Cyp1a1/2*, *Cyp2c9*, *Cyp2c19*, *Cyp2d6*, *Cyp2e1* and *Cyp3a* ↔, *Cyp2a5* ↑ Protein expression: CYP1A1/2, CYP2C9, CYP2C19, CYP2D6 and CYP3A ↔, CYP2A5 and CYP2E1 ↑ Enzyme activity: CYP1A1/2, CYP2A5, CYP2E1 and CYP3A ↑, CYP3C9, CYP2C19 and CYP2D6 ↔	62
Sprague-Dawley rat	f	19-week-old	HF diet (42.8% energy from fat)	mRNA expression: Cyp3a2, Abcb1a, Abcc2, Abcb11, Abcb4, Slco1a4 and Slc10a1 ↑, Abcc3 ↓ and Cyp7a1 ↔ Protein expression: only for Cyp3a2 ↑	63
C57BL/6J mouse	m	22-week-old	HFHS diet (54.4% fat und 28.3% carbohydrate)	mRNA expression*: Cyp1a1*, *Cyp1a2*, *Cyp2b10* and *Cyp2c29* ↑, *Cyp3a11* ↓ and *Cyp2d22* ↔	64
Tg-CYP2D6 mouse	m	26-week-old	HF diet (42% fat)	mRNA expression: *Cyp2d6, Cyp1a2* and *Cyp2c37* ↓, *Cyp2b10* and *Cyp3a11* ↑, *Cyp2e1* ↔ Protein expression: analysed only for CYP2D6 ↔ Enzyme activity: analysed only for CYP2D6 and CYP3A11 ↑	65
C57BL/6J mouse	m	17-week-old	HF diet (60% fat)	Enzyme activity: CYP2E1 ↑, CYP3A11, CYP3A25, CYP2C70, CYP1A2, CYP2C29, CYP4A12, CYP7A1 and CYP8b1 ↓	66
C57BL/6J mouse	m	20-week-old	HFHS diet (45% fat und 35% carbohydrate)	mRNA expression: *Cyp2c29*, *Cyp2c37* and *Cyp2c54* ↓, *Cyp2c50* ↔ In vitro formation of the active clopidogrel metabolite ↓	67
Sprague-Dawley rat und C57BL/6J mouse	m/f	Rat: 18-week-old and mouse: 16-week-old	Rat: HF diet (45% fat) mouse: HF diet (60% fat) and injection of streptozotocin to induce T2DM	mRNA expression: *Cyp1a2*, *Cyp3a2* and *Cyp2c11* ↓ (m, rat); *Cyp1a2* and *Cyp3a2* ↓ and *Cyp2c11* ↑ (f, rat) Protein expression: CYP2E1 ↔ (m and f rat), CYP3A1, CYP1A2 and CYP2C11 ↓ (m and f rat), CYP2D1 (m ↓ and f↔); CYP3A41 and CYP1A2 ↓ (m and f, mouse) and CYP2E1 ↑ (m and f, mouse); the diabetic male mice showed no difference compared with obese male mice, the formation of the active metabolite of lidocaine ↓ in rats as well as in mice	68
Human	m/f	29–64	Obese (BMI: 34–59) subjects who received daily doses of 20 mg atorvastatin	A significant negative correlation was found between BMI and the protein expression of CYP3A4 in liver as well as in small intestine. However, there was no significant correlations between BMI and the protein expression of OATP1B1 or MDR1 in the liver or small intestine. The oral clearance of atorvastatin showed a significant negative correlation with BMI	69
Human	m/f	18–62-year-old	Morbidly obese subject who received Roux-en-Y gastric bypass. Plasma pharmacokinetic was investigated by administration of a single oral dose of morphine sulphate (30 mg) to these patients before surgery	There was no correlation between morphine pharmacokinetic results and the jejunal protein expression of CYP3A4, P-glycoprotein, MRP2 and MRP3	70
Human	m/f	18–53-year-old	Morbidly obese subject who received gastric bypass surgery	Protein expression for CYP1A2 and GLUT4 showed a moderate correlation with BMI (only in jejunum)	71
Human	m/f	21–54-year-old	Normal weight controls, overweight and morbidly obese subjects who received either laparoscopic sleeve gastrectomy or Roux-end-Y-gastric bypass	Enzyme activity was analysed by using 5 drug probes (dextromethorphan, losartan, omeprazole, caffeine and paracetamol): CYP2D6 with two functional alleles ↑ in morbidly obese subjects, CYP3A4 ↓ in morbidly obese and normalised after bariatric surgery, CYP2C9 and CYP1A2 ↔	72
Human	m/f	27–57-year-old	Morbidly obese subject who received gastric bypass surgery	A significant negative correlation was found between BMI and hepatic CYP2B6, whereas there was no correlation between BMI and other hepatic P450 and intestinal P450 enzymes (in liver and jejunum)	73
Human	m/f	20–62-year-old	Subjects with a broad range of BMI (18–63), who enrolled in the COCKTAIL study	A significant negative correlation was found between BMI and hepatic CYP3A activity in liver biopsy samples	74

Increase (↑), decrease (↓) and no change (↔) compared with the control group and if not stated otherwise in liver tissue.

HO-1, haem oxygenase-1; NQO1, NAD(P)H quinone dehydrogenase; SULT, sulphotransferase family; UGT, UDP glucuronosyltransferase family; ABCB, ATP-binding cassette sub-family b; ABCC, ATP-binding cassette sub-family c; SLC, solute carrier family; SLCO, solute carrier organic anion transporter family; OATP1B1, organic anion transporting polypeptide 1B1; MDR, multi-drug resistance protein; MRP, multi-drug resistance-associated protein; GLUT4, glucose transporter type 4.

The mRNA and protein expression of CYP2E1 were analysed in liver, fat and kidney tissues of lean Zucker (+/+) rats which were either fed with a normal diet (ND) or a high-fat (HF) diet and obese Zucker (*fa*/*fa*) rats fed with ND (obese). Both ND and obese rats showed significantly higher levels of mRNA and protein expression for CYP2E1 in liver and fat tissues, but not in kidney tissue. In addition to the expression changes, an increase in the activity of CYP2E1 was measured after a single intravenous chlorzoxazone administration (Ref. 57). These results indicated an altered CYP2E1 activity in genetic as well as in diet-induced obesity models. In another study, the mRNA and protein expression of xenobiotic metabolising enzymes and transporters was analysed in liver and kidney samples from wild-type (C57BL/6) and obese mice (ob/ob). In the liver of obese mice, the expression of cytochrome P450 enzymes and efflux transporters was increased, whereas the expression of uptake transporters was decreased (Ref. 58). Jang *et al*. (Ref. 59) used an HF diet-induced obesity model and analysed the mRNA and protein expression of organic cation transporter OCT1 in the liver of C57BL/6 mice. They found an increased mRNA and protein expression for OCT1 in the HF diet group. In parallel to this, obese mice showed an increase in the plasma and hepatic concentration of metformin after intraperitoneal or intravenous metformin application.

In another study, the authors tested the effect of bamboo extract on hepatic metabolising enzymes in C57BL/6J mice fed with standard diet and HF diet. Their results indicated significantly higher activity for CYP1A2 and CYP3A11 in the HF diet and bamboo extract-fed groups. However, the protein expression of these two enzymes showed no significant difference compared with that found in the mice fed with standard diet (Ref. 60). The difference could be in the activity, which cannot be detected by expression measurements. Sawamoto and colleagues (Ref. 61) used lean Zucker (+/+) rats and obese Zucker (*fa*/*fa*) rats to understand their previous observation regarding the lower required maintenance dose of tacrolimus in obese patients compared with normal weight patients (BMI < 25) during a retrospective pharmacokinetic study. For this aim, a single-dose tacrolimus was administered either by intravenous injection or orally to the rats. The blood concentration–time profile of tacrolimus as well as the protein expression for CYP3A2 and P-glycoprotein in the liver and in the intestine were analysed. The results of this study demonstrated a significant reduction in protein expression of CYP3A2 in liver tissue of obese Zucker rats but not in intestine, whereas the expression of P-glycoprotein was increased in the intestine of obese Zucker rats but did not change in the liver. In addition, the blood concentration of tacrolimus was higher in obese Zucker rats after application via both routes, which indicated an increased bioavailability for tacrolimus and supported the patient study findings regarding lower maintenance dose of tacrolimus in obese patients.

Tomankova *et al*. (Ref. 62) analysed the activity and expression of cytochrome P450 enzymes in liver, small intestine and colon samples of mice in which repeated injections of monosodium glutamate had been used to induce obesity. Body weight, plasma insulin and leptin levels were significantly higher in the obese group compared with the control. The obese mice showed increased expression and activity of some liver cytochrome P450 enzymes (CYP1A1/2, CYP2A5, CYP2E1 and CYP3A). There was no difference between the groups in the small intestine and colon samples. Ghoneim *et al*. (Ref. 63) used an HF diet-induced obesity model and analysed the mRNA and protein expression of CYP3A2, the mRNA expression of CYP7A1 and the expression of various drug transporters in the liver of Sprague-Dawley rats. The expression of CYP3A2 was significantly increased in the obese group compared with the control animals, whereas the expression of CYP7A1 remained unchanged. The ATP-binding cassette (*Abc*) transporters 1a, c2, b11 and b4, the solute carrier transporter 10a1 and the solute carrier organic anion transporter 1a4 showed an increased mRNA expression, whereas the mRNA expression for *Abcc3* was significantly reduced.

In a study by Chiba and colleagues (Ref. 64), non-alcoholic fatty liver disease was induced in C57BL/6 mice by feeding a high-fat high-sucrose (HFHS) diet for 12 weeks. In addition to a standard diet group, the authors examined the protective effects of trans-resveratrol in HFHS diet mice. Animals in the HFHS group showed significantly higher body and liver weights, as well as increased accumulation of triglycerides and cholesterol. The mRNA expression of selected cytochrome P450 enzymes, except for *Cyp3a11*, was increased under the HFHS diet. The mRNA expression of *Cyp3a11* was reduced. Trans-resveratrol slightly reduced lipid accumulation in the liver but failed to restore cytochrome P450 enzyme expression. The change in hepatic metabolism was studied only by mRNA expression analysis, which alone does not adequately represent the level of protein expression and activity of these enzymes.

Ning and Jeong (Ref. 65) applied an HF diet to CYP2D6 humanised transgenic (Tg-CYP2D6) mice. Tg-CYP2D6 mice were fed either with HF diet or control diet for 18 weeks. Mice in the HF diet group had higher body and liver weights compared with the mice under control diet. Hepatic mRNA levels of *Cyp2d6* decreased slightly, but protein levels and activity did not differ between the diet groups. In addition to CYP2D6, the expression of other cytochrome P450 genes such as *Cyp1a2* and *Cyp2c37* was decreased in the HF diet group. The mRNA expression of *Cyp2b10* and Cyp3a11 was higher in the HF diet group.

Sadler *et al*. (Ref. 66) studied the effect of cigarette smoke on cytochrome P450 enzyme activity in normal weight mice and obese mice fed with an HF diet. These animals were exposed to either active or passive cigarette smoke or filtered air. Their results showed decreased activity for many cytochrome P450 enzymes in the HF diet group alone. Simultaneous exposure to cigarette smoke and HF diet significantly increased the activity of several cytochrome P450 enzymes.

In the study by Sun *et al*. (Ref. 67), C57BL/6J mice were fed with an HF diet for 10 weeks. This obesity mouse model was used to assess platelet aggregation and carotid arterial thrombosis in response to clopidogrel treatment. The results indicated impaired bioactivation of clopidogrel in obese mice because of downregulation of hepatic Cyp genes. The authors demonstrated reduced hepatic mRNA expression of *Cyp2c* genes, which is required for activation of the pro-drug clopidogrel.

In another study, Al Nebaihi and colleagues investigated the effect of an HF diet on cytochrome P450 enzymes in mice and rats and its influence on lidocaine metabolism (Ref. 68). Their results revealed reduced activity and expression of selected cytochrome P450 enzymes and reduction of the active lidocaine metabolite in mice and rats fed with the HF diet. These effects were normalised in rats by switching back to the standard.

Among the 12 studies described so far, five used a C57BL/6J mouse diet-induced obesity model. Of these, four analysed either the activity or expression of *Cyp1a2* and three that of *Cyp3a11*. Although Koide *et al*. (Ref. 60) determined increased activity for CYP1A2 and CYP3A11 in obese mice, Sadler and colleagues (Ref. 66) showed reduced activity of CYP1A2 and CYP3A11. Overall, these studies also demonstrated the importance of determining not only expression changes, but also enzyme activity, as alterations in metabolism were observed even with constant enzyme expression.

The listed studies employ a variety of ways to induce obesity. The cause of obesity is either genetic or diet induced and in the latter case the type of diet differs between studies. These differences might affect the grade of obesity as well as the manifestation of obesity-related comorbidities such as fatty liver, insulin intolerance and diabetes. Fatty liver and type 2 diabetes can affect hepatic health and metabolism, and a good characterisation of the progression of these comorbidities may contribute to a better understanding of the impact on liver metabolism.

The current literature regarding humans is usually limited to samples from morbidly obese patients without a healthy control group, as illustrated by the following studies. Ulvestad and colleagues (Ref. 69) analysed the protein expression of CYP3A4, the efflux transporter MDR1 and the organic anion transporter OATP1B1 in liver and small intestine biopsies from obese subjects. Their aim was to investigate the possible effect of the expression levels of these proteins on the pharmacokinetics of atorvastatin. They found a significant negative correlation between BMI values and protein expression of CYP3A4 in both tissues. In line with this, there was a negative correlation between BMI values and the oral clearance of atorvastatin. The protein expression of MDR1 or OATP1B1 in liver or intestine did not show a correlation with BMI values.

Lloret-Linares *et al*. (Ref. 70) collected jejunum tissue from morbidly obese subjects during Roux-en-Y gastric bypass surgery to study the association between obesity and the protein expression of CYP3A4, P-glycoprotein and multi drug resistance-associated proteins 2 and 3 (MRP2 and MRP3). In addition, they investigated the link between oral morphine pharmacokinetics in obese subjects and the jejunal expression of selected drug metabolising enzymes or drug transporters. For this, the oral pharmacokinetics of morphine (30 mg single dose) was analysed before the surgery. However, the pharmacokinetic parameters of morphine did not show any association with the jejunal protein expression for the selected drug metabolising enzymes or drug transporters. Overall, the protein expression in jejunum showed a large difference between individuals.

In another study, protein expression of metabolising enzymes in jejunal tissue samples from 28 morbidly obese subjects undergoing bariatric surgery was quantified (Ref. 71). The results showed a significant influence of BMI, gender and smoking on the expression of some metabolising enzymes. The protein expression of CYP1A2 was positively correlated with BMI (Ref. 71). Rodríguez-Morató and colleagues (Ref. 72) used drug probes (Karolinska cocktail including dextromethorphan, losartan, omeprazole, caffeine and paracetamol) to analyse the four major cytochrome P450 enzymes (CYP2D6, CYP3A4, CYP2C9 and CYP1A2) in normal weight, overweight and morbidly obese subjects before as well as 1 and 6 months after bariatric surgery. The estimated CYP3A4 activity was lower in morbidly obese subjects compared with normal weight controls and bariatric surgery helped to normalise its activity. The activity of CYP2D6 was higher in morbidly obese subjects than in normal weight controls. BMI did not have any effect on the activity of CYP2C9 and CYP1A2. Krogstad and colleagues (Ref. 73) collected liver and small intestine biopsy samples from 20 obese patients undergoing gastric bypass surgery after 3 weeks of low-energy diet. Seven cytochrome P450 enzymes in these samples were examined. The results showed interindividual variability in the activity of the P450 enzymes. CYP2B6 activity correlated negatively with BMI. In a recent study from Krogstad and colleagues (Ref. 74), liver biopsies from 36 individuals with a broad range of BMI (18–63) were analysed for an ex vivo activity of drug metabolising enzymes (CYP3A, CYP2B6, CYP3c8, CYP2D6, CYP2C9, CYP2C19 and CYP1A2). Their findings demonstrated a significant negative correlation between BMI and CYP3A activity in liver tissue. For the other CYP enzymes, there was no correlation. However, the enzyme activities for some of the selected CYPs (CYP2B6, CYP2C8, CYP2D6, CYP2C19 and CYP1A2) were not quantifiable because of either low enzyme activity or insufficient amount of tissue.

Overall, current literature explicitly aiming at metabolic enzymes in humans is limited to only a few cytochrome P450 enzymes. However, some additional published studies which used probe drugs to investigate drug clearance are reviewed elsewhere (see Refs 75, 76). Drug clearance is influenced by alterations in expression/activity of drug metabolising enzymes, but also by other factors such as distribution within the body or blood flow, and therefore might not enable a direct conclusion regarding expression or activity of metabolic enzymes. Therefore, we focused in this review on articles that studied the expression and/or activity of metabolic enzymes.

The results from animal studies are ambiguous (Table 5). No consistent effect was observed for any of the investigated cytochrome P450 enzymes, except for CYP2B10 which was only assessed in mice at the RNA level. One reason may be that there are large differences in nutritional conditions and pathophysiology (fatty liver, type 2 diabetes, etc.) between the various rodent models.

**Table 5.** Summary for the effect of obesity on the most common studied cytochrome P450 enzymes in liver tissue from rodent obesity models of Table 4

Type of CYP	Number of studies	Effect of obesity		
		mRNA expression	Protein expression	Enzyme activity
CYP1A2	Mouse (4*x*) and rat (2*x*)	Mouse (2*x*): ↔ ↑ rat (2*x*): ↓	Mouse (2*x*): ↔ rat (0*x*)	Mouse (3*x*): ↑↑↓ rat (0*x*)
CYP2B10	Mouse (3*x*)	Mouse (3*x*): ↑	Mouse (0*x*)	Mouse (0*x*)
CYP2D6	Mouse (2*x*)	Mouse (2*x*): ↓↔	Mouse (2*x*): ↔	Mouse (0*x*)
CYP2E1	Mouse (4*x*) and rat (2*x*)	Mouse (2*x*): ↓ ↔ rat (1*x*): ↑	Mouse (3*x*): ↓↑↑ rat (2*x*): ↑↔	Mouse (2*x*): ↑ rat (1*x*): ↑
CYP3A2	Rat (3*x*)	Rat (2*x*): ↓↔	Rat (2*x*): ↔	Rat (0*x*)
CYP3A11	Mouse (5*x*)	Mouse (3*x*): ↑↓↔	Mouse (2*x*): ↔↓	Mouse (3*x*): ↓↑↑

Increase (↑), decrease (↓) and no change (↔) compared with the control group.

Extrapolation of animal data to humans can pose a great challenge because of cross-species differences in the expression of drug metabolising enzymes (Ref. 77). Although in vivo models are very useful to study complex disease models, additional research with modern cell culture techniques such as organoids or ‘human on a chip’ would provide further insights. Overall, well-designed studies to elucidate the effects of obesity on xenobiotic metabolism and the possible causes and affected enzymes or proteins are still needed.

## Conclusions, knowledge gaps and future directions

**Fig. 1.**
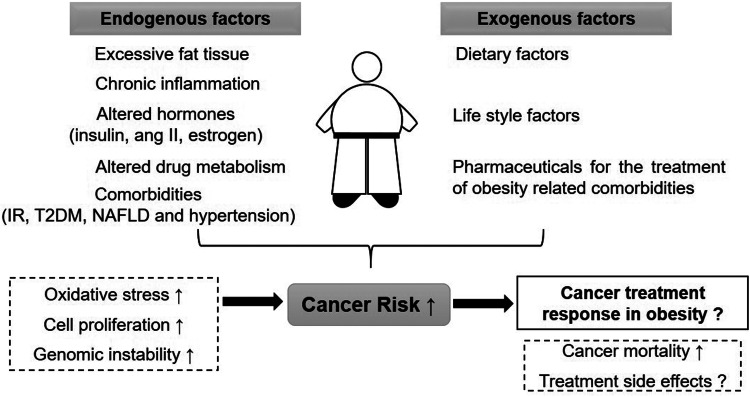
Overview on the relationship between obesity, cancer risk and cancer therapy. ang II: angiotensin II, IR: insulin resistance, T2DM: type 2 diabetes mellitus, NAFLD: non-alcoholic fatty liver disease.

The relationship between obesity, cancer risk and cancer survival is highly complex. Figure 1 gives an overview of endogenous and exogenous factors in obesity and their influence on cancer risk as well as cancer therapy. There are various additional factors that can modify the outcome; for example, the grade of obesity, existing comorbidities, sex and age of the patient, type of cancer and selected therapy approach.

The existing literature shows convincingly that obesity is a risk factor for several cancer types. However, the underlying mechanisms are not always clear. Elevated DNA damage is meanwhile well documented in obesity, although the causations are not only BMI dependent, but also linked to chronic inflammation (Ref. 78), oxidative stress (Refs 17, 79, 80, 81) and altered hormones such as insulin (Refs 82, 83). The vast majority of DNA damage can be eliminated by DNA repair mechanisms. Although there are some indications that DNA repair activity might be impaired in obesity, more research is needed to understand the role of DNA repair in the elevated cancer risk in obesity. In addition to all above specified endogenous cancer risk factors, exogenous risk factors can also play an important role in the obesity-related cancer risk. For example, lifestyle factors such as physical activity and nutritional preferences can contribute to cancer risk but can also be modified and play an important role in cancer prevention. Dietary preferences can modify the food-borne carcinogen exposure. For example, consuming ultra-processed food may increase the exposure to food additives and processing contaminants (Ref. 84). Exposome research is a growing field which offers an approach to define the effects of endogenous and exogenous exposures to human health (Ref. 85). With increasing numbers of studies in this area, we will be able to define the external risk factors for obese individuals better.

Obesity may alter the susceptibility of an individual and thus modulate the response to an exposure. Regarding cancer therapy, poor prognosis, reduced survival and increased side effects are documented for obese patients in some studies. Dose adjustment in chemotherapy is critical for therapy success, but also for reducing side effects. Increased toxicity can lead to therapy interruptions and reduction of therapy success. Body surface area is one of the common methods for dose adjustment, but it might not be the best approach for obese patients. The American Society of Clinical Oncology (ASCO) recommends using full weight-based chemotherapy to treat obese patients with cancer, but also recommends a fixed dose only for some chemotherapeutics such as carboplatin. ASCO also states that further research is needed to understand the alterations in pharmacokinetic and pharmacogenetic parameters of chemotherapeutics for obese cancer patients (Ref. 52). We still have insufficient information on the influence of obesity on hepatic drug metabolism and drug clearance. An in-depth understanding on the role of obesity in xenobiotic metabolism can also help to adjust risk assessment for carcinogenic exposures to the increasing prevalence of obesity. Therefore, future studies addressing this question are essential.
